# Production and Characterization of Cosmetic Nanoemulsions Containing *Opuntia ficus-indica* (L.) Mill Extract as Moisturizing Agent

**DOI:** 10.3390/molecules20022492

**Published:** 2015-02-02

**Authors:** Renato Cesar de Azevedo Ribeiro, Stella Maria de Andrade Gomes Barreto, Elissa Aarantes Ostrosky, Pedro Alves Rocha-Filho, Lourena Mafra Veríssimo, Márcio Ferrari

**Affiliations:** 1 College of Pharmacy, Federal University of Rio Grande do Norte, Rua Gustavo Cordeiro de Farias, s/n, Petrópolis, Natal 59012-570, RN, Brazil; E-Mails: recar_89@hotmail.com (R.C.A.R.); smagbarreto@gmail.com (S.M.A.G.B.); aranteselissa@gmail.com (E.A.O.); lourenamafra@yahoo.com.br (L.M.V.); 2 College of Pharmaceutical Sciences of Ribeirão Preto, University of São Paulo, Avenida Zeferino Vaz, s/n, Monte Alegre, Ribeirão Preto 14040-903, SP, Brazil; E-Mail: pedranjo@fcfrp.usp.br

**Keywords:** nanoemulsions, *Opuntia ficus-indica*, non-invasive methods, emulsions, formulation/stability, skin barrier

## Abstract

This study aimed to produce and characterize an oil in water (O/W) nanoemulsion containing *Opuntia ficus-indica* (L.) Mill hydroglycolic extract, as well as evaluate its preliminary and accelerated thermal stability and moisturizing efficacy. The formulations containing 0.5% of xanthan gum (FX) and 0.5% of xanthan gum and 1% of *Opuntia ficus-indica* MILL extract (FXE) were white, homogeneus and fluid in aspect. Both formulations were stable during preliminary and accelerated stability tests. FX and FXE presented a pH compatible to skin pH (4.5–6.0); droplet size varying from 92.2 to 233.6 nm; a polydispersion index (PDI) around 0.200 and a zeta potential from −26.71 to −47.01 mV. FXE was able to increase the water content of the stratum corneum for 5 h after application on the forearm. The O/W nanoemulsions containing 1% of *Opuntia ficus-indica* (L.) Mill extract presented suitable stability for at least for 60 days. Besides, this formulation was able to increase the water content of stratum corneum, showing its moisturizing efficacy.

## 1. Introduction

Emulsions are a dispersion of an immiscible liquid into another stabilized by the presence of a third compound: the surfactant [1]. Among these formulations we can highlight a class of emulsions called nanoemulsions, characterized by their small particle size (50–500 nm) [2]. Nanoemulsions are thermodynamically unstable and kinetically stable systems [3]. The very small droplet size can reduce the occurrence of creaming, sedimentation and flocculation during storage. This happens due to the capacity of Brownian motion to reduce the force of gravity [4]. Ostwald ripening is the main instability process on nanoemulsions, because the small droplet size results in a higher driving force (Laplace pressure) [5,6]. The preparation method is very important for the formation and stability of nanoemulsions [7,8,9]. These systems can be prepared by high or low emulsification energy methods [10]. High-energy approaches utilize mechanical devices (microfluidizers, high pressure homogenizers or ultrasonic methods) that generate intense forces capable of forming very fine oil droplets. Low energy methods involve complex interfacial hydrodynamic phenomena and depend on the system composition properties [11].

Nanoemulsions are attractive systems for use in the cosmetics, pharmaceutical, food and other industries due to their low amount of surfactant, higher stability against coalescence, lack of toxicity or irritant characteristics, low viscosity, good appearance, and versatility of formulation as foams, creams, liquids and sprays [7,12]. Nanoemulsions are particularly useful systems for cosmetics because the small droplet size ensures a closer contact with the stratum corneum (SC), increasing the amount of active compound reaching the desired site of action. Besides, nanoemulsions can carry actives into the skin improving the skin layer penetration, thus enhancing efficacy [13].

The human skin has an important function providing a barrier between the internal contents of the organism and the external physical, chemical, and biological environment. This barrier protects against hazardous microorganisms, toxic chemicals, ultraviolet radiation and water loss [14].

Dry skin is a common problem among the population. Its main characteristic is a rough appearance and peeling, a phenomenon called xerosis. The barrier function and maintenance of the water content are dependent on the SC that provides mechanical protection and acts against water loss and the passage of external solutes. Changes in the stacking of corneocytes and delipidation are causes of cutaneous dryness leading to decreased skin flexibility and harming the skin’s protective barrier [15].

Moisturizers are one of the most important classes of cosmetic products due to their preventive action against xerosis and delaying of premature ageing and for their use to help dermatological therapies in a wide variety of skin disorders [16]. Moisturizers may act by an occlusive mechanism, impairing evaporation of skin moisture by forming an epicutaneous lipidic film that prevents water loss, as is the case with oils and lipids; or as humectants, *i.e.*, glycerin, urea, sodium pyrrolidone carboxylic acid; which act by attracting water from the other layers of the epidermis to the stratum corneum [17]. Another two moisturizing mechanism are active hydration by rearranging the stratum corneum and aquaporin formation. The aquaporins are transmembrane proteins which form water channels and facilitate water flux through the cell plasma membrane thus being important to maintain a constant water content in viable epidermis [18,19].

Herbal products have gained increase popularity and the sales of herbal cosmetics are expanding nowadays [20]. *Opuntia ficus-indica* (L.) Mill is a plant that grows in arid areas. The semiarid region accounts for 12% of the extension of Brazil, where the Caatinga biome, an exclusively Brazilian bioma, prevails [21]. This plant has a huge socioeconomic importance to the semiarid region [22,23]. Its cladodes are mainly composed by water (80%–95%) and carbohydrates (3%–7%) [22]. Phenolic components (kaempherol and quercetin) and carbohydrates (galacturonic acid, glucose, rhamnose and arabinose) are some of the substances present in the chemical composition of *Opuntia ficus-indica* (L.) Mill and the cosmetic industry has interest in these substances as moisturizers and anti-aging products [24,25,26].

The aim this research was to produce and characterize an oil in water (O/W) nanoemulsion containing *Opuntia ficus-indica* (L.) Mill hydroglycolic extract, as well as evaluate its thermal stability and its moisturizing efficacy.

## 2. Results and Discussion

### 2.1. Nanoemulsion Formulations

Many variables are important when formulating a cosmetic product. The choice of emollients needs attention because their physico-chemical properties like molecular weight, chain length and polarity will affect sensorial properties like spreadability, final cosmetic sensorial properties and oleosity [27]. Caprylic/Capric Triglyceride, Ethylhexyl Palmitate and C12–15 Alkyl Benzoate were chosen due to their sensory and skin conditioning properties, and also for their dry and non-greasy touch.

The type of emulsion formed (W/O or O/W) is mainly dependent on the surfactant system used in the emulsification process, and the amount of each phase that could influence this process to form a nanoemulsion [28]. Nanoemulsions are formulations with high water content [29], so we tested formulations with water contents varying from 50% to 92%. Shakeel *et al*. [30] and Teo *et al*. [31] state that a high percentage of surfactant content may cause skin irritation. Sevcíková *et al*. [32] obtained nanoemulsions by varying the surfactant level between 3.0% to 5.0%, but they suggested that it is possible to obtain nanoemulsions using 1.5% to 10% of surfactant, what justifies the variation of surfactant concentration from 3% to 10% in our study [32].

All emulsions prepared were white, homogeneous and had a fluid aspect. With the aim of choosing the best formulation samples were submitted to centrifugation and high temperature exposure one day (24 h) after preparation of the nanoemulsions, as these are the most used stress situations to quickly evaluate the stability of an emulsion [33,34].

Emulsions were considered stable since no creaming, flocculation or phase separation were observed during the available time [35]. Samples F2, F3 and F4 showed some modification 24 h after preparation and phase separation happened five days later at 45 °C and 75% ± 5% relative humidity (RH). Samples F5, F6, F7 and F8 remained normal after 24 h, but showed some modifications after centrifugation and/or five days at 45 °C. After all tests, sample F1 showed a homogeneous and fluid aspect with a slightly bluish-white color, which can be caused by the Tyndall effect, typical of nanoemulsions, and was the only sample that showed no modifications.

The use of high molecular weight polymers, that are shear thinning (pseudoplastic) and give a very high viscosity at low shear rates, is one of most common procedures for reducing instability phenomena in the continuous phase [36]. Xanthan gum was thus added to the water phase to increase the stability of nanoemulsions and decrease the degree of nanoparticle aggregation [37]. The addition of xanthan gum to F1 (FX) resulted in a system presenting white color, homogeneous and with a fluid aspect, but presenting higher consistency.

The obtained extract presented a viscous and clear liquid appearance with a greenish-yellow color and characteristic odor as described by Dasmasceno [24]. FX (a formulation with 0.5% of Xanthan Gum) and FXE (a formulation with 0.5% of Xanthan Gum and 1% of *Opuntia ficus-indica* MILL extract) were submitted to preliminary and accelerated stability tests. The addition of extract to the aqueous phase did not change the macroscopic aspect of the formulations.

### 2.2. Preliminary Stability Tests

Stability tests are necessary due to their predictive capacity. For that, formulations are submitted to situations aimed to accelerate changes that may occur under market conditions. Results of stability tests are not absolute, but have a good probability of success [38].

Both formulations (FX and FXE) remained homogenous after 3000 rpm centrifugation, with a white color and macroscopic homogeneity, as shown in Table 1. The absence of phase separation is a positive factor in the stability evaluation, seeing that when phase separation occurs all characteristics of emulsions are affected [39]. Phase separation occurred at temperatures over 60 °C during thermal stress. This result shows the instability of these nanoemulsions on high temperatures. Komaiko and McClements [40] obtained nanoemulsions by spontaneous emulsification and by the phase inversion method, using non-ionic surfactants, and observed instability at high temperatures too, concluding that nanoemulsions produced by low energy methods and with these non-ionic surfactants are likely to be thermally sensitive due to the properties of the surfactants [40].

After freeze-defrost cycles, nanoemulsions showed a homogeneous aspect. FX samples presented a pH value about 5.50 ± 0.05 and FXE 5.49 ± 0.01, which were not statistically different (*p* < 0.05) from the values measured before the cycles (Table 1). These pH values are suitable for skin care products, where values should be between 4.5–6.0 [41], therefore, these nanoemulsions were considered suitable for use.

Determination of electrical conductivity is the most common procedure to investigate phase inversion. When this process occurs on an O/W emulsion, the conductivity decreases strongly [36]. This parameter helps to identify the type of nanoemulsions because large conductivity values, like values obtained in our study, characterize O/W nanoemulsions [42]. Conductivity values decreased for both formulations, as described in Table 1. Bernardi *et al*. [43] observed conductivity changes in nanoemulsions but considered the samples stable due to the absence of signs of macroscopic destabilization of the systems. It is reported that it is difficult to assess the stability only using conductivity as there is no linear relation between this parameter and the instability process [43].

**Table 1:** Results of preliminary stability tests of nanoemulsions with and without *Opuntia ficus-indica* (L.) Mill extract.

Parameters	FX	FXE
**After 24 h**		
Centrifugation	N	N
pH value	5.62 ± 0.04	5.53 ± 0.02
Electrical conductivity (µS/cm)	676.0 ± 3.0 *	689.4 ± 4.7 *
Droplet size (nm)	142.6 ± 2.3 *	139.1 ± 2.8 *
PDI	0.26 ± 0.01	0.23 ± 0.01
Zeta potential (mV)	−38.10 ± 1.69 *	−42.98 ± 0.88
**After TS**		
pH value	N/A	N/A
Electrical conductivity (µS/cm)	N/A	N/A
Droplet size (nm)	N/A	N/A
PDI	N/A	N/A
Zeta potential (mV)	N/A	N/A
**After FDC**		
pH value	5.55 ± 0.05	5.49 ± 0.01
Electrical conductivity (µS/cm)	457.7 ± 33.6 *	510.7 ± 3.1 *
Droplet size (nm)	160.6 ± 3.3 *	148.8 ± 1.6 *
PDI	0.21 ± 0.02	0.26 ± 0.01
Zeta potential (mV)	−47.01 ± 1.64 *	−42.58 ± 0.99

Notes: FDC = Freeze-Defrost Cycles; TS = Thermal Stress; N = Normal; N/A = Not evaluated, PDI = Polidispersion Index; FX = Sample containing 0.5% Xanthan Gum; FXE = Sample containing to 0.5% Xantham Gum and 1% *Opuntia ficus-indica* extract. * *p* < 0.05 compared to the initial time.

It is known that the smaller the droplet size is the better emulsion stability is. This happens because Brownian motion becomes more significant than the force of gravity [44,45]. Determination of particle size and zeta potential are the most common methods employed to assess the stability of nanoemulsions, because particle size interferes with flocculation and coalescence phenomena [7]. Particle size is an important parameter to define application and stability. The droplet size showed a slight increase after the end of the cycles (12th day) as demonstrated in Table 1, however, droplet radius remained below the 100 nm size that characterizes a nanoemulsion according to McClements [3].

A lower PDI value (near zero) indicates a monodisperse droplet population, whereas a PDI value closer to 1 (one) indicates a wide range of droplet sizes [46]. Despite the fact the droplet size slightly increased after this test, the PDI value remained around 0.2, indicating the homogeneity of the droplet population in both formulations.

A zeta potential value between |−25 mV| and |−30 mV| is already enough to create an energy barrier between the droplets thus avoiding coalescence [47]. Maruno and Rocha-Filho [48] found significant negative zeta potentials using nonionic surfactants, and associated this phenomenon with some chemical properties of the polyoxyethylene chains in the surfactants used [49]. The zeta potentials of the formulations prepared were above −30 mV before and after the test for both formulations, so the formulations were considered stable enough for the accelerated stability tests.

### 2.3. Accelerated Stability Tests

In the development of a cosmetic product, it is important to consider the appearance of the formulation, physical properties and stability [33,50]. At 25 °C and 45 °C, there was a statistically significant (*p* < 0.05) decrease of the pH value. According to Gallarate [51] this change could be associated with degradation of some oil phase components yielding free fatty acids (Table 2). Despite this statistically significant change of pH values, they remained in the ideal range of the skin (between 4.5–6.0) ensuring the stability of formulations, and this pH change did not modify their visual aspect. Bernardi *et al*. [43] observed a decrease of pH values of O/W nanoemulsions containing rice bran oil after storage at 40 ± 2 °C for 90 days and associated that fact to a probable hydrolysis process caused by temperature, but pH values also remained in an acceptable range for the skin in that study [43].

**Table 2:** Results of accelerated stability tests of nanoemulsions with and without *Opuntia ficus-indica* (L.) Mill extract.

	FX				FXE			
	Initial 24 h	After 60 Days			Initial 24 h	After 60 Days		
		4 °C	25 °C	45 °C		4 °C	25 °C	45 °C
**Macroscopic characteristics**	**N**	**N**	**N**	**N**	**N**	**N**	**N**	**N**
**pH**	5.67 ± 0.03	5.68 ± 0.03	5.62 ± 0.03 *	5.42 ± 0.02 *	5.62 ± 0.02	5.63 ± 0.02	5.56 ± 0.02 *	5.32 ± 0.21 *
**Particle size (nm)**	126.3 ± 5.9	136.5 ± 10.8	185.9 ± 52.4 *	117.9 ± 8.8	119.0 ± 7.4	121.6 ± 8.8	233.6 ± 30.1 *	92.17 ± 6.1 *
**PDI**	0.264 ± 0.030	0.231 ± 0.071	0.349 ± 0.052	0.260 ± 0.020	0.257 ± 0.038	0.251 ± 0.020	0.305 ± 0.126	0.228 ± 0.044
**Zeta potential (mV)**	−28.60 ± 10.33	−44.35 ± 4.07 *	−36.53 ± 12.94	−43.28 ± 4.72 *	−26.71 ± 6.67	−41.71 ± 4.55 *	−39.77 ± 5.99 *	−41.21 ± 6.02 *

Notes: * *p*-valor < 0.05; N—Normal; PDI—Polydispersion index; FX is the formulation with 0.5% Xanthan Gum; FXE is the formulation with 0.5% Xanthan Gum and 1% of *Opuntia ficus-indica* L. Mill extract.

It is very important that nanoemulsions remain physically stable throughout their shelf life, and for that no or a minimal change in the particle size distribution is necessary [11]. The emulsion stability is closely related to the droplet size distribution. A large droplet size may enhance Ostwald ripening what makes droplet size increase and can lead to coalescence and creaming [50].

**Figure 1:**
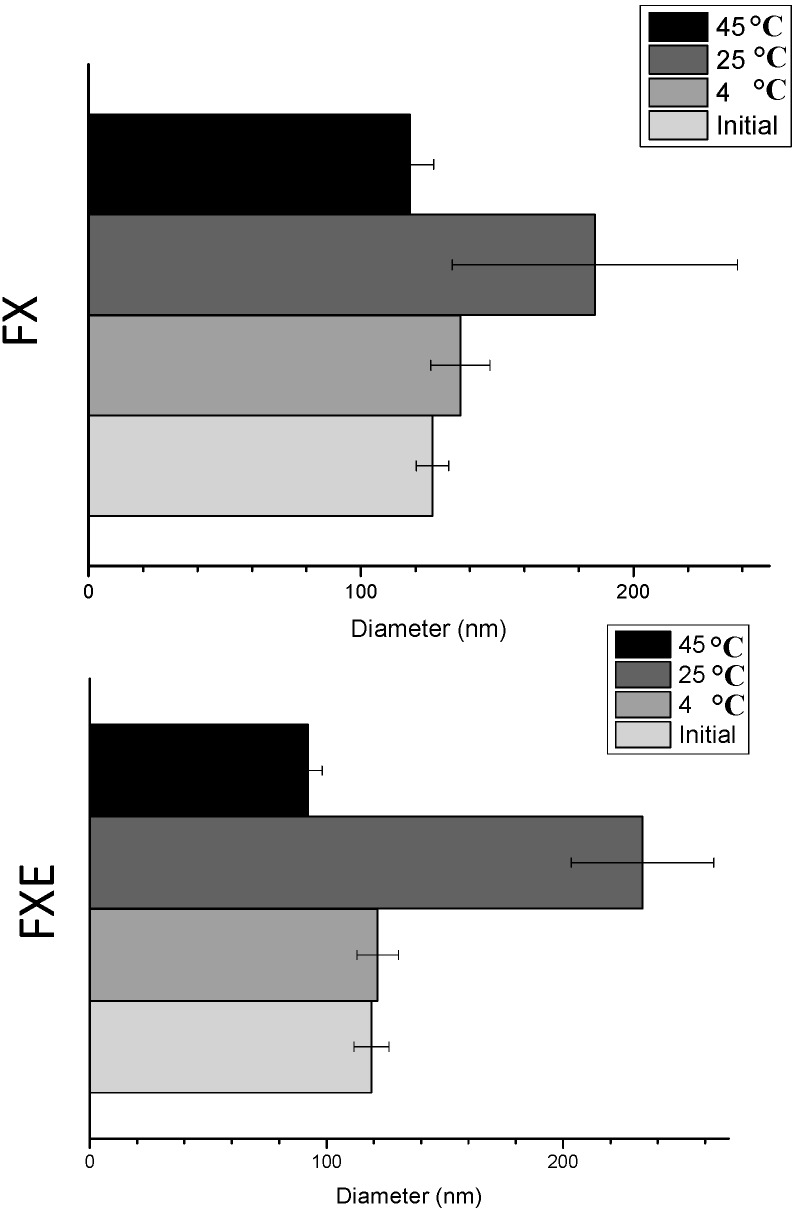
Droplet size graphic of accelerated stability tests of nanoemulsions with and without *Opuntia ficus-indica* (L.) Mill extract.

**Figure 2:**
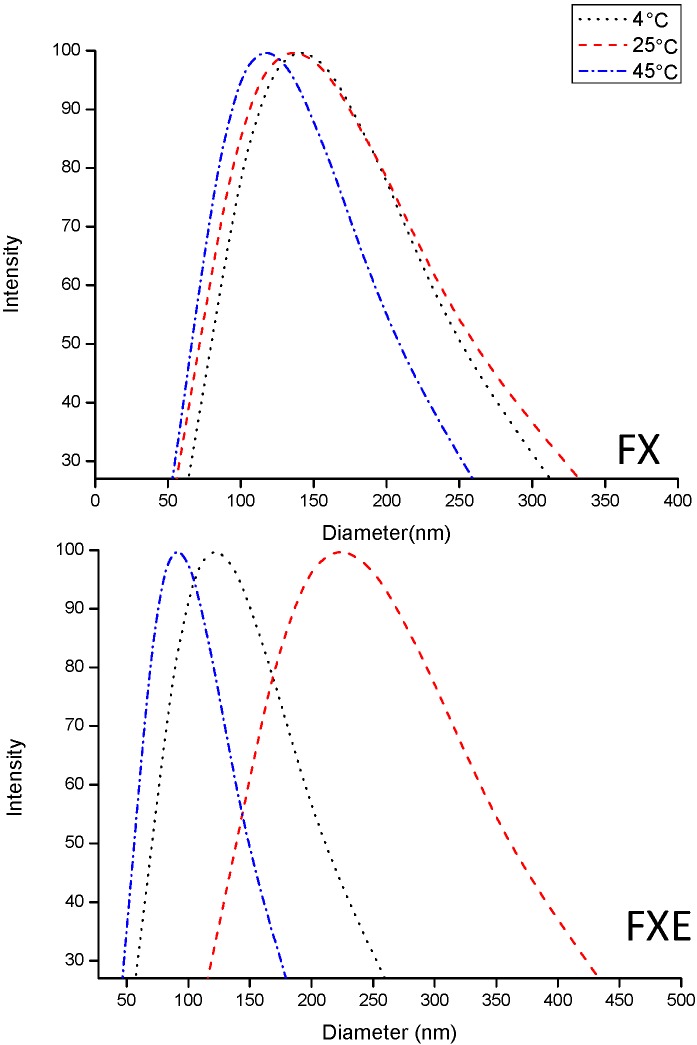
Dynamic Light Scattering (DLS) size distribution of stable samples (FX and FXE) after 60 days.

The relation between storage temperature and emulsion stability is well known. Samples stored at 4 °C maintained the same droplet size until the end of 60 days (Table 2, Figure 1). Ševčíková *et al*. [28] also reported better results for samples stored at 4 °C at which minimal particle size changes happen, compared to samples stored at higher temperatures [28]. Samples stored at 45 °C (except FX) and 25 °C showed significant changes in particle size after 60 days (Figure 1 and Figure 2). Despite these changes, the nanoemulsions did not show any macroscopic instability processes and their particle size remained in the nanoscale range, taking into account that the droplet size range of nanoemulsions is 100–500 nm [4]. Like in our study, Ševčíková *et al*. [32] produced stable nanoemulsions in which an increase in droplet size from 140 nm (25th day) to 250 nm (28th day) at 25 °C storage temperature was observed.

PDI values varied from 0.228 to 0.349 showing no statistically significant (*p* < 0.05) difference (Table 2). Changes in particle size but not in PDI also happened in other studies as found by Saberi, Fang and McClements [6].

**Figure 3:**
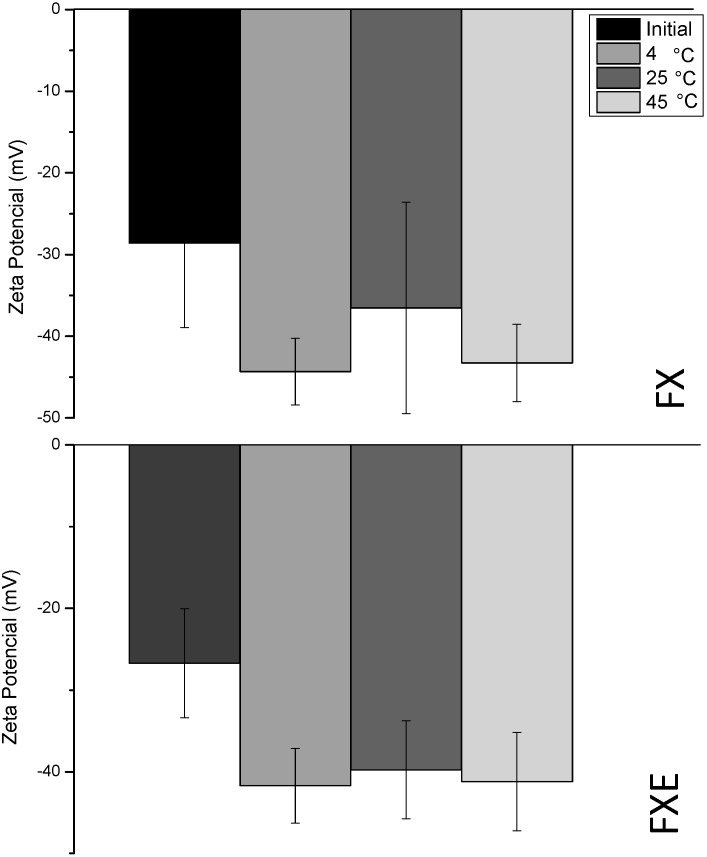
Zeta potential graphic of accelerated stability tests of nanoemulsions with and without *Opuntia ficus-indica* (L.) Mill extract.

Zeta potential values were |−26.7 mV| and |−28.6 mV| for FX and FXE in the initial measurement, respectively (Table 2, Figure 3). During the storage, zeta potential increased, in modulus, with the accommodation and stabilization of the system. After 60 days this parameter was close to |−40 mV| which indicates good physical stability of the dispersed systems, because high zeta potential values ensure repulsion of droplets [36,52]. Bazylińska *et al*. [53] observed zeta potential values to range between |−42 mV| and |+47 mV| in anionic and cationic kinetically stable nanoemulsions and attributed that stability to these high values of zeta potential, because this parameter is responsible for the stability of colloidal dispersions and indicates the degree of repulsion between similarly charged particles in the dispersion. Decreases in zeta values are related to a greater flocculation tendency, which was not the case of the nanoemulsions in this study [49].

The FX and FXE samples were considered stable because they remained inside acceptable parameters, what means inside the nanoscale droplet size range and macroscopically stable.

### 2.4. Evaluation of in Vivo Moisturizing Properties

Moisturizing products are evaluated by the increase of water content in the stratum corneum and by the decrease in TEWL. SC hydration measurements are widely employed to verify the moisturizing effects of topical products [54]. The measurement of skin hydration using the Corneometer^®^ CM 825 (Courage & Khazaka Electronic GmbH, Köln, Germany) is based on the capacitance effect. The dry SC is a dielectric medium and its dielectric properties change with changes in moisture content [55].

**Figure 4:**
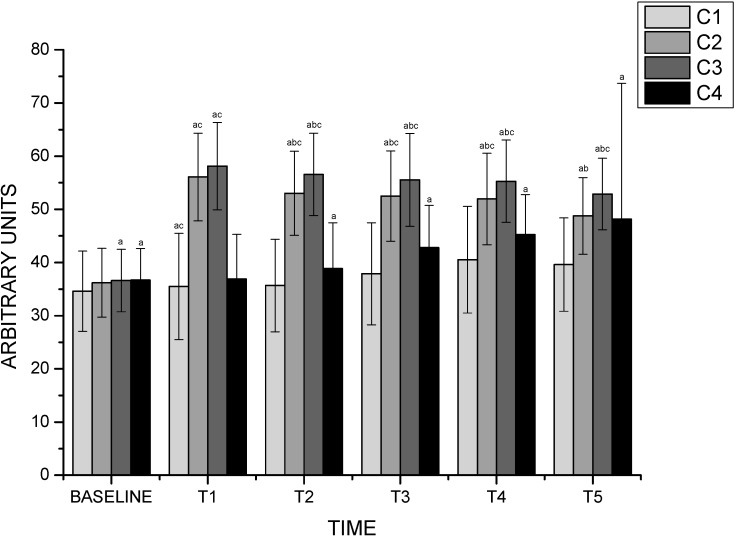
Water content values of the stratum corneum measured by Corneometer^®^ CM 825 before and after 1, 2, 3, 4 and 5 h application of formulations.

Notes: a = *p*-value < 0.05 for ANOVA + Tukey post-test *vs.* negative control. b = *p*-value < 0.05 for ANOVA + Tukey post-test *vs*. C2 and C3. c = *p*-value < 0.05 for ANOVA + Tukey post-test *vs*. positive control. C1—Negative control; C2—vehicle; C3—Vehicle + 1% *Opuntia ficus-indica* L. Mill extract; and C4—Positive control (commercial moisturizer). T1—After one hour; T2—After two hours; T3—After three hours; T4—After four hours; T5—After five hours.

A moisturizer product prevents SC dehydration when the environmental humidity reaches very low values. When the water content is adequate, the skin maintains its barrier function, flexibility and healthy appearance [54]. At the beginning of analysis (basal), the mean volunteer hydration was under 40 arbitrary units. This profile is related to a dry skin according to the standard scale of this equipment [55]. Our results show a statistically significant increase (*p* < 0.05) of skin hydration after the application of our formulation on the skin and this effect lasted for 5 h (Figure 4).

After product application, we can observe a significant statistically difference (*p* < 0.05) between the FX and FXE formulations after the second hour, proving the moisturizer potential of *Opuntia ficus-indica* (L.) Mill extract. Some reports in the literature show that carbohydrate derivatives exert a moisturizing effect, so we can affirm that *Opuntia ficus-indica* (L.) Mill extract has a moisturizing effect due to its large amount of carbohydrates (galacturonic acid, glucose, rhamnose and arabinose) [25,56]. Dal’belo *et al*. [17] studied formulations containing or not different amounts of aloe vera extract. In this study a significant difference between all formulations containing the extract was observed compared to vehicle formulation after 2 and 3 h after application, proving the extract’s moisturizing effect and not only the effect of the vehicle [17].

Nanotechnological products show some improvement on their characteristics when compared to other products. Damasceno [24] observed a moisturizing tendency in conventional emulsions containing *Opuntia ficus-indica* (L.) Mill extract. The use of nanoemulsions in our study allowed the observation of a better moisturizing effect performance when compared to conventional emulsions with the same extract added. According to Morganti [13] this could be associated with the better properties of nanoemulsions due to the small droplet size that facilitates uniform deposition on the skin and more permeability through the skin layers [13]. Besides, a commercial moisturizer was used as a positive control in this study. This parameter allow verifying the course of the experiment because the commercial moisturizer showed a statistically significant result for hydration of the stratum corneum after the second hour and maintained this moisturizing effect for at least 5 h after application.

We noted no statistically significant alteration of TEWL and barrier function compared with basal measurements, meaning that transepidermal water loss did not decrease (Table 3). However, our formulations were able to maintain the water loss under 10 g/mm^2^·h for all volunteers, which characterizes very healthy skin as directed by Courage-Khazaka [55]. Thus, our formulation was able to maintain skin health. Experimental formulations with 1% tetraisopalmitoyl ascorbic acid (IPAA) were developed by Maia-Campos *et al*. [57] and showed a similar profile. There was a significant increase of stratum corneum water content but the skin barrier function was not altered [57].

**Table 3:** Transepidermal water loss values before and 1, 2, 3, 4 and 5 h after application of formulations.

	C1	C2	C3	C4
BASAL	6.06 ± 1.71	6.40 ± 1.60	6.91 ± 1.09	9.06 ± 2.24
T1	6.47 ± 1.70	6.49 ±1.88	7.57 ± 1.67	8.13 ± 1.78
T2	6.95 ± 2.21	6.74 ± 1.34	6.66 ± 1.88	7.66 ± 2.04
T3	7.10 ± 1.47	6.87 ± 1.36	7.48 ± 1.60	8.31 ± 2.34
T4	7.00 ± 1.80	7.15 ± 1.53	6.85 ± 1.85	7.74 ± 2.06
T5	7.37 ± 2.36	7.16 ± 2.21	7.55 ± 2.32	8.38 ± 2.57

Notes: C1—Negative control; C2—vehicle; C3—Vehicle + 1% *Opuntia ficus-indica* L. Mill extract; and C4—Positive control (commercial moisturizer). T1—After one hour; T2—After two hours; T3—After three hours; T4—After four hours; T5—After five hours. TEWL in g/mm^2^·h.

Dal’Belo *et al*. [17] noted that formulations supplemented with freeze-dried aloe vera extract significantly increased the water content of the stratum corneum but did not change the TEWL when compared with the vehicle, like in our study [17]. This was related to the composition of freeze-dried aloe vera extract that presented a rich hygroscopic mono- and polysaccharides content and the amino acids histidine, arginine, threonine, serine, glycine and alanine, that have a humectant mechanism of action. Like freeze-dried aloe vera extract, *Opuntia ficus-indica* (L.) Mill extract has no occlusive properties but can increase the retention of water on the skin surface, thus we can hypothesize that formulations containing *Opuntia ficus-indica* (L.) Mill extract could act by a humectant mechanism and not by an occlusion mechanism [19].

Gaspar *et al*. [56] evaluated the moisturizer efficacy of formulations containing *Saccharomyces cerevisiae* extract and vitamins and found similar results. Even though the formulation caused an increase in stratum corneum hydration, no changes were observed in the TEWL leading to the conclusion that this formulation also has a moisturizing effect due to a humectant mechanism [56].

Damasceno [24], observed a decrease in TEWL up to four hours after application of a gel-cream containing the same concentration of *Opuntia fícus-indica* hydroglycolic extract indicating an occlusive effect of these formulations. This proves that the vehicle could influence the moisturizing process mechanism due to the differences in system characteristics between a gel-cream and a nanoemulsion coupled to the presence of a polymer that could make the formulation more occlusive, justifying the decrease on TEWL with the gel-cream used by Damasceno and no decrease using our studied nanoemulsions.

## 3. Experimental Section

### 3.1. Materials

The surfactants used were Polysorbate 80 (HLB = 15) and Sorbitan Oleate (HLB = 4.5) (Croda do Brasil Ltda., Campinas, SP, Brazil). The oil phase was composed of Capric/Caprilic Triglycerides, Ethylhexyl Palmitate, C12–15 Alkyl Benzoate (Croda do Brasil Ltda., Campinas, SP, Brazil), Paraffinum Liquidum (Êxodo Científica, Hortolândia, SP, Brazil), Phenoxyethanol (and) Caprylyl Glycol (Ashland Inc., São Paulo, SP, Brazil) and BHT (Galena Química e Farmacêutica, Campinas, SP, São Paulo, Brazil). The water phase was composed by Disodium EDTA (DEG Importação de Produtos Químicos, Sorocaba, SP, Brazil); Xanthan Gum (CP Kelco Brasil, Limeira, SP, Brazil) and distilled water.

### 3.2. Preparation of Opuntia ficus-indica (L.) Mill Extract

The cladodes of *Opuntia ficus-indica* were supplied by the Agricultural Research Corporation of Rio Grande do Norte, Brazil (5°17'27.71''S, 36°16'27.63''W). A voucher specimen was deposited at the Herbarium of the Federal University of Rio Grande do Norte under number UFRN-16600. The hydroglycolic extract was obtained from fresh cladodes. A 20:80 (w/w) water/propylene glycol mixture was used as extraction vehicle. To perform the extraction a 1:3 (w/w) plant-solvent ratio was used. The cladodes were washed with distilled water, cut into small pieces and macerated for 12 h. Subsequently, the resultant mixture was percolated under a flow of 2 mL/min to obtain the extract [24].

### 3.3. Preparation and Formulations

The oil and water phases were heated separately to 75 ± 2 °C. The oil phase was added to the aqueous phase, followed by agitation at 11.000 rpm (IKA, Ultra Turrax Mod. T18 basic, Staufen, Germany) during five minutes. Samples were placed in an ice bath under mechanical agitation (IKA, mod. Digital RW 20, Staufen, Germany) at 500 rpm during 2 min [49]. The *Opuntia ficus-indica* (L.) Mill hydroglycolic extract was added and the system was stirred for 2 min (Table 4).

The Hydrophilic Lipophilic Balance (HLB) required by the oil phase was theoretically calculated according to the values provided by manufactures of oil phase compounds. Different samples of emulsions were prepared varying the surfactant, oil and water amounts, as detailed on Table 4.

Emulsions were evaluated macroscopically 24 h after formation, centrifuged at 3000 rpm (Fanen, mod. 206 BL, Guarulhos, SP, Brazil) during thirty minutes and samples were stored at 45 ± 2 °C and 75% ± 5% RH (Nova Etica, mod. 520-CLDTS 150, Vargem Grande Paulista, SP, Brazil) for 5 days. Stable formulation (F1) was added to Xantham Gum 0.5% and *Opuntia ficus-indica* (L.) Mill extract 1% was added into it (FXE) or not (FX) (Table 4).

**Table 4:** Nanoemulsion compositions (%, w/w).

COMPONENTS	F1	F2	F3	F4	F5	F6	F7	F8	FX	FXE
BHT	0.1	0.1	0.1	0.1	0.1	0.1	0.1	0.1	0.1	0.1
Caprylic/Capric Triglyceride	2.0	4.0	8.0	1.0	1.0	2.0	3.0	1.0	2.0	2.0
Ethylhexyl Palmitate	2.0	4.0	8.0	1.0	1.0	2.0	3.0	1.0	2.0	2.0
C12–15 Alkyl Benzoate	1.0	2.0	4.0	0.5	0.5	1.0	1.5	0.5	1.0	1.0
Paraffinum Liquidum	5.0	10.0	20.0	2.5	2.5	5.0	7.5	2.5	5.0	5.0
Phenoxyethanol (and) Caprylic Glycol	1.0	1.0	1.0	1.0	1.0	1.0	1.0	1.0	1.0	1.0
Sorbitan Oleate	4.7	4.7	4.7	4.7	2.4	2.4	2.4	1.4	4.7	4.7
Polysorbate 80	5.3	5.3	5.3	5.3	2.6	2.6	2.6	1.6	5.3	5.3
Disodium EDTA	0.1	0.1	0.1	0.1	0.1	0.1	0.1	0.1	0.1	0.1
Xanthan Gum	---	---	---	---	---	---	---	---	0.5	0.5
*Opuntia ficus-indica* (L.) Mill extract	---	---	---	---	---	---	---	---	---	1.0
Distilled Water	78.8	68.8	48.8	83.8	88.8	83.8	78.8	90.8	78.3	77.3

Notes: FX = Sample added to 0.5% Xanthan Gum; FXE = Sample added to 0.5% Xanthan Gum and 1% *Opuntia ficus-indica* extract.

### 3.4. Preliminary Stability Tests

Centrifugation test: centrifugation was performed on 24 h-old preparations at 3000 rpm (Fanen, mod. 206 BL, Guarulhos, SP, Brazil) for 30 min at room temperature [48]. The appearance, homogeneity and organoleptic characteristics were evaluated by macroscopic analyses [48].

Thermal stress: emulsions were submitted to a heated thermostatic bath (Logen Scientific, mod. LSBMLS 2006-2, São Paulo, SP, Brazil) set for the temperature range of 40 to 80 °C, with temperature increase at intervals of 5 °C, and holding at each temperature for 30 min. The organoleptic characteristics, pH value determination and electrical conductivity measures were obtained to evaluate the formulations before and at the end at 80 °C, after the natural cooling of the samples at room temperature (25 ± 2 °C) [33].

Freeze-defrost cycles: samples were subjected to 4 ± 2 °C/24 h (Consul, Facilite Frost free 300 L, Joiville, SC, Brazil) and then 45 ± 2 °C/24 h and 75% ± 5% RH (Nova Etica, mod. 520-CLDTS 150, Vargem Grande Paulista, SP, Brazil), thus completing a cycle [33].

The organoleptic characteristics, pH value determination, electrical conductivity measures, droplet size, PDI and zeta potential were evaluated before and after preliminary stability tests [33,49].

pH value determination: 1.0 g of nanoemulsion and 9.0 g of distilled water were placed in a test tube and homogenized. The pH value (Hanna Instruments, mod. HI 21, Tamboré, SP, Brazil Brazil) was determined by inserting the electrode directly into the aqueous dilution 1:10 (w/w) of the sample [33].

Electrical conductivity determination: electrical conductivity measures (Logen Scientific, mod. CD-300-K1) were evaluated at a temperature of 25 ± 2 °C by inserting the electrode directly into the sample [33].

Determination of particle size and zeta potential: The mean particle size and zeta potential of nanoemulsions were measured by Dynamic Light Scattering (DLS) and phase analysis light scattering, respectively (Brookhaven ZetaPALS, Holtsville, NY, USA). The formulations were diluted with distilled water by 200-fold before the measurement [58].

### 3.5. Accelerated Stability Tests

The samples considered stable by preliminary tests were stored under different conditions: 4 ± 2 °C (Consul, Facilite Frost free 300 L, Joinville, SC, Brazil); 25 ± 2 °C (room temperature), and 45 ± 2 °C and 75% ± 5% RH (Nova Etica, mod. 520-CLDTS 150, Vargem Grande Paulista, SP, Brazil). The samples were maintained under these conditions for 60 days. The macroscopic analyses (appearance, homogeneity and organoleptic characteristics), pH value determinations, droplet size, PDI and zeta potential were evaluated at different time intervals (24 h after preparation of formulations and on 7th, 30th and 60th days) [48,49].

### 3.6. Evaluation of Mosturizing Eficacy in Vivo

This study was approved by the Research Ethics Committee of the University of Cuiabá (2012-041). A total of 18 volunteers with a range of ages from 20 to 65 years old, with no history of previous skin disease were included in this study after having given their written informed consent. This study was designed as a one-sided blind, placebo-controlled study. The volunteers were instructed not to use any cosmetic products for two weeks before and on the day of the experiment, except cleaning products like soap [57,59]. Prior to all measurements, volunteers were left in the room for at least 30 min in order to allow full skin adaptation to the room’s temperature (21 ± 2 °C) and relative humidity (60% ± 5%) [57,59].

Four sites (9 cm^2^ each) on the volunteers’ forearm skin were chosen (C1 to C4): one (C1) serving as the control where only measurements were taken, and the others (C2 to C4), where different formulations were applied. C2 had FX formulation (vehicle), C3 had the formulation containing 1.0% of *Opuntia ficus-indica* hydroglycolic extract—FXE, and a commercial moisturizer was used as positive control in C4. All formulations were applied to the surface of the skin at a dose of 2 mg/cm^2^ with a light massage of approximately 10 s [57,59].

Stratum corneum moisture content was determined by noninvasive biometrical measurements using a skin capacitance meter (Corneometer^®^ CM 825-Courage & Khazaka Electronic GmbH, Köln, Germany) whereas transepidermal water loss was determined by an evaporimeter (Tewameter^®^ TM 300-Courage & Khazaka Electronic GmbH, Köln, Germany). TEWL values were registered for 2 min following a 30 s period of equilibration of the probe on the skin. The baseline measurements (control area—region which received no formulation) of skin hydration were taken after at least 30 min of acclimatization under standard climatic conditions. Skin capacitance and TEWL were determined before, and at 1, 2, 3, 4 and 5 h after a single application of the formulations to areas C2-C4 and to the control field without formulation (C1). For skin capacitance measurement, nine readings were taken in each field. For TEWL evaluation, one reading in each field was taken [57,59].

## 4. Conclusions

The interest and investment in the cosmetic area concerning the advent of nanotechnology has motivated this study that contributes to the field of moisturizing cosmetics by producing a stable nanoemulsified system, that can improve the performance of cosmetic products when compared to conventional techniques and emulsified products. Beyond that, the study collaborated with the cosmetic market trends that look for products with vegetal raw materials and contributed to produce a cosmetic containing a regional Brazilian Caatinga biome product thus adding value to this region’s vegetation. The O/W nanoemulsion containing 1% of *Opuntia ficus-indica* (L.) Mill hydroglycolic extract could increase water content in the stratum corneum for 5 h and showed a significant difference when compared to vehicle formulation (FX). The high moisturizing capacity of FXE was probably related to the chemical composition of *Opuntia ficus-indica* (L.) Mill which is rich in carbohydrates (galacturonic acid, glucose, rhamnose and arabinose), suggesting a humectant mechanism. Overall, these results indicate that the produced oil-in-water nanoemulsion was stable and had moisturizing efficacy, proving to be a product with potential in the cosmetic area.
